# Serum CXCL9 and CCL17 as biomarkers of declining pulmonary function in chronic bird-related hypersensitivity pneumonitis

**DOI:** 10.1371/journal.pone.0220462

**Published:** 2019-08-01

**Authors:** Yoshihisa Nukui, Takashi Yamana, Masahiro Masuo, Tomoya Tateishi, Mitsuhiro Kishino, Ukihide Tateishi, Makoto Tomita, Takehiro Hasegawa, Takashi Aritsu, Yasunari Miyazaki

**Affiliations:** 1 Department of Respiratory Medicine, Tokyo Medical and Dental University, Bunkyo-ku, Tokyo, Japan; 2 Department of Diagnostic Radiology, Tokyo Medical and Dental University, Bunkyo-ku, Tokyo, Japan; 3 Department of Clinical Research Center, Tokyo Medical and Dental University, Bunkyo-ku, Tokyo, Japan; 4 Sysmex Corporation, Nishi-Ku, Kobe, Japan; Medical Center - University of Freiburg, GERMANY

## Abstract

The clinical course of chronic hypersensitivity pneumonitis (HP) with fibrosis is similar to that of idiopathic pulmonary fibrosis (IPF). Current research is expected to identify biomarkers effective in predicting the deterioration of lung function in a clinical setting. Our group analyzed the relationships between the following parameters in chronic bird-related HP: patient characteristics, serum markers, lung function, HRCT findings, BALF profiles, and the worsening of lung function. We also analyzed serum levels of CXCL9, CCL17, and Krebs von den Lungen 6 (KL-6) as serum markers. Patients showing declines in vital capacity (VC) of over 5% at 6 months after first admission were categorized as the “decline group”; the others were categorized as the “stable group.” The serum level of CCL17 and the percentage of BALF macrophages were significantly higher in the decline group compared to the stable group. Serum levels of CXCL9 and CCL17 were significant variables in a multivariate logistic regression analysis of factors associated with VC decline. Patients with a chemokine profile combining lower serum CXCL9 and higher serum CCL17 exhibited significantly larger VC decline in a cluster analysis. Higher serum CCL17 and lower serum CXCL9 were important predictors of worsening lung function in patients with chronic bird-related HP.

## Introduction

Hypersensitivity pneumonitis (HP) is an immune-mediated lung disease triggered by the inhalation of a wide variety of antigens [1]. The clinical presentation of HP is traditionally classified into acute and chronic types. Acute HP strikes in acute episodes and can be successfully cured by allergen avoidance. Chronic HP is categorized into two subgroups, (recurrent type and insidious type, according to the clinical features) [2]. Patients with the recurrent type experience repeated acute episodes of mild exertional dyspnea, cough, and low-grade fever. Patients with the insidious type suffer from chronic, slowly progressing respiratory disease without acute episodes. From 70 to 80% of acute HP cases are summer-type HP caused by *Tricosporon*, while about 50% of chronic HP cases are bird-related [2].

The clinical course of chronic HP with fibrosis is similar to that of idiopathic pulmonary fibrosis (IPF). Lung function rapidly deteriorates in some patients with chronic HP. Reports on the prognosis of chronic HP are scanty. Among patients with chronic HP, those with a usual interstitial pneumonia (UIP) pattern have a poorer prognosis and higher incidence of acute exacerbation (AE) compared to patients with fibrotic nonspecific interstitial pneumonia (fNSIP) and a cellular NSIP (cNSIP)/organizing pneumonia (OP) pattern [3, 4]. The extent of fibroblastic foci could be a useful predictor of mortality in chronic HP with a UIP pattern [5]. In another study, CT findings of airspace consolidation and honeycombing were found to be predictive of mortality in chronic HP [6]. From another perspective, the levels of exposure to avian antigen have been found to be related to disease progression and prognosis in chronic bird-related HP [7, 8]. To date, there are no established biomarkers to predict the worsening of lung function. One such biomarker, however, is expected to emerge for patients with chronic HP.

We conducted a retrospective study to evaluate the predictors of worsening lung function. First, we analyzed the relationships between the following factors: patient characteristics, serum markers, lung function, high-resolution computed tomography (HRCT) findings, bronchoalveolar lavage fluid (BALF) profiles, and worsening lung function. Then, drawing from previous evidence that Th2-predominant immune response may play an important role in the development of lung fibrosis in chronic HP, we also analyzed serum levels of CXCL9 (Th1 chemokine), CCL17 (Th2 chemokine), and Krebs von den Lungen 6 (KL-6) [9].

## Methods

### Subjects

Eight hundred and eight patients were hospitalized at our center for the treatment of interstitial lung disease between January 2004 and December 2013. Chronic HP was diagnosed in this population based on previously described clinical, radiological, and histological criteria (details are provided in the online supplement) [10]. Patients underwent the inhalation provocation test with an avian antigen to support and refine the bird-related HP diagnosis [11]. Sixty-six patients were diagnosed with chronic bird-related HP based on positive results in the inhalation provocation test. Five out of the 66 patients were excluded: one with a medical history of connective tissue disease and four whose samples had not been stored. Finally, 61 patients diagnosed with chronic bird-related HP were recruited into this retrospective study. None of the patients with chronic bird-related HP had medical histories of atopic dermatitis or bronchial asthma, and none were receiving treatments at the time of diagnosis. In parallel, 50 healthy volunteers (HV) were evaluated as controls. The study conformed to the Declaration of Helsinki and was approved by the Medical Research Ethics Committee of Tokyo Medical and Dental University (M2015-577). Informed consent was obtained for all patients in the following method. In adherence with the Ethical Guidelines for Medical and Health Research Involving Human Subjects, information on the study implementation was made public to ensure that the subjects had the opportunity to withdraw their consent at any time by publishing on the web site. Therefore, written informed consent from the enrolled patients was waived by the ethics committee.

### Study design

Medical records, pulmonary function tests (PFTs), HRCT findings, BALF findings, and analyzed blood sample data of patients with chronic bird-related HP were reviewed as baseline data upon first admission to our institution. Follow-up data on PFTs and AEs were also obtained. The follow-up period was the period from the first admission to our institution to the final observation (July 15, 2017). The criteria of Kondoh were used to define an AE of chronic bird-related HP [12].

### High-resolution computed tomography

Three thin-slice HRCT images at the levels of the aortic arch, carina, and inferior pulmonary vein were extracted. The respective slices from the right and left lungs were reviewed independently by two observers (M.M., a pulmonary specialist, and M.K., a chest radiologist) who had no knowledge of the patients’ clinical information. The fibrosis area and ground grass opacities (GGO) were respectively assigned a fibrosis score and GGO score by Kazerooni’s method [13]. Reticulation, centrilobular nodules, consolidation, and emphysema were separately quantified as proportions of lung parenchyma between 0% and 100%, and censored at 5%. Traction bronchiectasis (TBE) was scored as previously reported [14]: grade 0 = none, 1 = mild, 2 = moderate, 3 = severe, based upon the most severely affected airways in that pattern. The global score was calculated as the mean of the six zones composed of each slice from the right and left lungs. Averages of all of the values assessed by the two observers were calculated for each subject.

### Pulmonary function tests

The PFT data included the vital capacity (VC) and the diffusing capacity for carbon monoxide (DL_CO_). Declines in the PFTs 6 months after the first admission were determined by calculating the differences in the PFT measurements (ΔVC, Δ%VC, ΔDL_CO_, and Δ%DL_CO_). The changes in PFT results could be analyzed in 48 out of 61 patients with chronic bird-related HP. Previous reports demonstrated that declines in forced vital capacity of over 5% at 6 months were associated with mortality in patients with IPF [15, 16]. Referring to these results, patients who showed declines in VC of over 5% and of less than 5% 6 months after the first admission were respectively assigned to the “decline group” and the “stable group” in this study.

### Bronchoalveolar lavage

BAL was performed using three 50-ml aliquots of sterile 0.9% saline. The cellular profile of BALF was determined by counting 200 cells in a cytospin smear with Wright’s stain. The lymphocyte phenotypes were analyzed by flow cytometry using monoclonal antibodies for CD4 and CD8. The BALF profiles of forty-four patients with chronic bird-related HP could be analyzed in the study. The CXCL9 and CCL17 BALF levels of thirty-one out of 44 patients could be analyzed by the following method, because their samples had been stored.

### Measurement of CXCL9, CCL17 and KL-6

CXCL9, CCL17, and KL-6 serum levels were measured by a fully automatic immunoanalyzer, the HISCL-5000 (Sysmex Corp., Hyogo, Japan). CXCL9 and CCL17 BALF levels were also measured by the HISCL-5000.

### Analysis of corticosteroid efficacy

For analysis of corticosteroid efficacy, we calculated the differences in VC and serum KL-6 between the start of corticosteroid treatment and at 6 months after treatment (ΔVC ^treatment^ and ΔKL-6 ^treatment^). KL-6 at the first day of corticosteroid treatment and at 6 months after treatment were extracted from the medical records. In this analysis, KL-6 was measured by a chemiluminescence enzyme immunoassay using the Lumipulse KL-6 Fujirebio (Fujirebio Co., Tokyo, Japan).

### Statistical analysis

Data were described as the median and interquartile ranges. Two-tailed *P* values of less than 0.05 were considered significant. The two groups were compared using the Mann-Whitney U test or Fisher’s exact test. Correlation coefficients including inter-observer variation for the extent of various abnormalities were obtained using Spearman’s correlation coefficient test. The most appropriate cutoff values were defined by receiver operating characteristic (ROC) analysis using the Youden index, and the area under the curve (AUC) values were calculated. Then, obtained cutoff and AUC values were validated using a leave-one-out cross-validation method. Briefly, one sample was omitted, whereas the AUC and cutoff values were calculated on the remaining samples. This was repeated until every sample was left out once. We verified the AUC and cutoff values from all samples comparing with the mean values obtained leave-one-out method. Cumulative survival curves and AE-free interval curves were constructed with the Kaplan-Meier method. Comparisons of the cumulative survival rate and the cumulative rate of being free from AE between two groups was based on the log-rank test. Factors associated with declines in VC of over 5% at 6 months after first admission were evaluated by logistic regression analysis. Univariate and multivariate logistic regression analyses were used to investigate predictors of declines in VC of over 5% at 6 months after first admission. Multivariate analysis was performed using variables with univariate *P* values of less than 0.2. The univariate and multivariate analyses were performed using SPSS Statistics version 22.0 (IBM Corp., Chicago, IL, USA). In cluster analysis, serum CXCL9 and CCL17 levels were converted to logarithm and calculated those averages and standard deviations. The calculated values were standardized and analyzed in cluster analysis. An unsupervised hierarchical cluster analysis was conducted using Cluster 3.0 (University of Tokyo Human Genome Center). The cluster analysis was performed by complete linkage based on Euclidean distance. The other analyses were performed using GraphPad Prism version 5.0 (GraphPad Software Inc., San Diego, CA, USA).

## Results

### Patient characteristics and clinical findings

The patient characteristics, CXCL9, CCL17, and KL-6, serum levels, and PFT results are shown in Table 1 and S1 Fig. The HRCT findings and BALF profiles are shown in S1 and S2 Tables. With regard to the HRCT findings, the inter-observer correlations in the assessed levels of the various radiologic abnormalities were statistically significant (*r* = 0.521–0.893, all *P* < 0.001) (S3 Table). The patients with chronic bird-related HP were significantly older compared to the healthy volunteers (*P* < 0.001). The CXCL9, CCL17, and KL-6 serum levels were significantly higher in the patients with chronic bird-related HP than in the healthy volunteers (CXCL9; chronic bird-related HP vs. HV, 19.3 (13.2–35.6) vs. 10.5 (7.6–15.6) pg/ml, *P* < 0.001. CCL17; 543.1 (336.0–767.6) vs. 274.4 (199.7–338.9) pg/ml, *P* < 0.001. KL-6; 1182 (552–1965) vs. 184 (148–240) U/ml, *P* < 0.001.).

**Table 1:** Patient characteristics.

Characteristic	Chronic bird-related HP	HV
	(n = 61)	(n = 50)
Gender		
Male	33	30
Female	28	20
Age, yr.	64.0 (56.5–71.0) **	47.0 (41.0–54.0)
Pack years	5.0 (0.0–28.0)	ND
Surgical lung biopsy	20	ND
Histological patterns	UIP 11/ fNSIP 8/ cNSIP1	
Serum CRP, mg/dl	0.1 (0.1–0.2)	ND
Serum KL-6, U/ml	1182 (552–1965) **	184 (148–240)
Serum CXCL9, pg/ml	19.3 (13.2–35.6) **	10.5 (7.6–15.6)
Serum CCL17, pg/ml	543.1 (336.0–767.6) **	274.4 (199.7–338.9)
A-aDO_2_, mmHg	17.4 (11.7–25.3)	ND
PFTs		
VC, L	2.17 (1.60–2.83)	ND
%VC	78.7 (65.4–86.2)	ND
DLco, ml/min/mmHg	9.78 (6.26–12.45)	ND
%DLco	55.9 (39.6–66.2)	ND
Acute exacerbation	18 (30%)	ND
Treatment^§^	45 (74%)	ND

Data are given as numbers or medians and interquartiles.

* *P* < 0.05 vs. HV.

** *P* < 0.01 vs. HV.

ND: not determined, HP: hypersensitivity pneumonitis, HV: healthy volunteers, UIP: usual interstitial pneumonia, fNSIP: fibrotic nonspecific interstitial pneumonia, cNSIP: cellular nonspecific interstitial pneumonia, KL-6: Krebs von den Lungen 6, A-aDO_2_: alveolar-arterial oxygen difference, PFTs: pulmonary function tests, VC: vital capacity, DL_co_: diffusing capacity of the lung for carbon monoxide.

^§^: Treatment during observation periods. Twenty-five patients were treated with a corticosteroid and immunosuppressants, and 20 patients were only treated with a corticosteroid. Ten patients were treated with antifibrotic agents in addition to a corticosteroid or immunosuppressants.

### Comparison of clinical findings between the decline and stable groups

We compared the clinical findings of the decline and stable groups to investigate the predictors of worsening lung function (Table 2). Serum levels of CCL17 and the percentage of macrophages in BALF were significantly higher in the decline group compared to the stable group (CCL17; decline group vs. stable group, 676.1 (569.9–916.0) vs. 411.4 (200.4–605.7) pg/ml, *P* < 0.001. BALF macrophage; decline group vs. stable group, 84.4 (75.3–92.7) vs. 65.1 (37.0–82.3) %, *P* = 0.015). On the other hand, there was no statistically significant difference in the pulmonary lung function test at the base line between the two groups.

**Table 2:** Comparison of clinical findings between the decline and stable group in patients with chronic bird-related HP.

	Decline group	Stable group	*P*
Number	14	34	
Age, yr.	60.5 (51.8–69.0)	64.5 (57.3–72.0)	0.216
Gender (Male / Female)	8 / 6	14 / 20	0.313
Pack years	10.0 (0.0–28.0)	0.0 (0.0–25.3)	0.387
Treatment during first 6 months	3	13	0.262
Acute exacerbations	6	9	0.315
Serum biomarkers			
CXCL9, pg/ml	15.9 (10.0–21.3)	23.3 (15.4–38.5)	0.068
CCL17, pg/ml	676.1 (569.9–916.0)	411.4 (200.4–605.7)	<0.001**
KL-6, U/ml	592 (473–2126)	1239 (663–1776)	0.358
PFTs			
VC, L	2.4 (1.6–2.7)	2.0 (1.6–2.8)	0.847
%VC	78.1 (61.9–81.6)	77.0 (65.5–86.0)	0.547
ΔVC, L	-0.26 (-0.36–-0.17)	0.03 (-0.01–0.18)	<0.001**
Δ%VC	-8.15 (-11.50–-5.50)	1.40 (-0.88–5.86)	<0.001**
HRCT findings			
GGO score	0.67 (0.33–1.42)	1.00 (0.50–1.42)	0.327
Fibrosis score	1.17 (0.96–1.46)	1.00 (0.83–1.42)	0.651
Reticulation, %	6.25 (4.17–8.13)	5.42 (3.33–6.67)	0.262
Centrilobular nodules, %	0.00 (0.00–2.50)	0.00 (0.00–1.25)	0.739
Consolidation, %	1.67 (0.21–3.33)	0.42 (0.00–1.25)	0.057
Emphysema, %	0.00 (0.00–0.42)	0.00 (0.00–0.00)	0.335
TBE grade	0.50 (0.42–0.83)	0.50 (0.33–0.75)	0.387
BALF			
Macrophage, %	84.4 (75.3–92.7)	65.1 (37.0–82.3)	0.015*
Lymphocytes, %	9.9 (3.6–19.6)	18.3 (9.2–44.8)	0.068
Neutrophils, %	0.9 (0.7–4.0)	2.6 (0.8–19.5)	0.229
Eosinophils, %	1.5 (0.4–1.7)	1.1 (0.6–1.9)	0.859
CD4/CD8 ratio	2.6 (1.5–4.3)	3.3 (2.0–8.3)	0.490

Data are given as numbers or medians and interquartiles.

* *P* < 0.05.

** *P* < 0.01.

Patients who showed declines in VC of over 5% and of less than 5% 6 months after the first admission were respectively assigned to the “decline group” and the “stable group”.

KL-6: Krebs von den Lungen 6, PFTs: pulmonary function test, HRCT: high-resolution computed tomography, GGO: ground grass opacity, TBE: traction bronchiectasis, BALF: bronchoalveolar lavage fluid.

### Logistic regression analysis of factors associated with VC decline

A logistic regression analysis of the following factors was conducted to determine which were associated with VC decline: PFTs, serum biomarkers, HRCT findings, BALF profiles, and history of treatment over the 6-month period following first admission (Table 3). All chronic bird-related HP patients were instructed to avoid not only direct exposure (breeding birds) but also unrecognized exposure (feather products, wild birds, and breeding of birds by neighbors). We assumed that there was no substantial difference among patients regarding the status of antigen avoidance. A multivariate analysis was performed using variables with univariate *P* values of less than 0.2 and clinically relevant covariates such as age and gender. BALF macrophages were excluded from the variables in the multivariate analysis because they were strongly correlated with the BALF lymphocytes (*r* = -0.813, *P* < 0.001). The CXCL9 and CCL17 serum levels were significant variables in the multivariate analysis (CXCL9; odds ratio 0.864, 95% CI 0.749–0.997, *P* = 0.045. CCL17; odds ratio 1.006, 95% CI 1.001–1.011, *P* = 0.022).

**Table 3:** Logistic regression analysis of factors associated with VC decline.

	Odds Ratio	95% Confidence interval	*P*
**Univariate analysis**			
Serum biomarkers			
CXCL9	0.968	0.921–1.017	0.191
CCL17	1.004	1.001–1.007	0.007**
KL-6	1.000	0.999–1.000	1.000
PFTs			
VC	1.002	0.431–2.331	0.996
%VC	0.980	0.940–1.022	0.346
HRCT findings			
GGO score	0.744	0.294–1.878	0.531
Fibrosis score	1.514	0.354–6.479	0.576
Reticulation	1.005	0.904–1.117	0.932
Centrilobular nodules	0.942	0.761–1.166	0.583
Consolidation	1.519	0.990–2.230	0.056
Emphysema	0.920	0.743–1.139	0.272
TBE grade	1.571	0.248–9.947	0.632
BALF			
Macrophage	1.068	1.005–1.134	0.033*
Lymphocytes	0.941	0.877–1.009	0.090
Neutrophils	0.398	0.848–1.038	0.217
Eosinophils	0.933	0.660–1.320	0.696
CD4/CD8 ratio	0.886	0.687–1.142	0.350
Treatment during first 6 months	0.441	0.103–1.882	0.269
**Multivariate analysis**			
CXCL9	0.864	0.749–0.997	0.045*
CCL17	1.006	1.001–1.011	0.022*

Covariates with *P* < 0.2 in univariate analysis and clinical relevant covariates such as age and gender were included in multivariate analysis (Logistic regression). Because macrophages of BALF had strong correlation with lymphocytes of BALF (*r* = -0.813, *P* < 0.001), macrophages of BALF was excluded from variables of multivariate analysis.

* *P* < 0.05.

** *P* < 0.01.

HP: hypersensitivity pneumonitis, KL-6: Krebs von den Lungen 6, PFTs: pulmonary function test, HRCT: high-resolution computed tomography, GGO: ground grass opacity, TBE: traction bronchiectasis, BALF: bronchoalveolar lavage fluid.

### Relationship between the serum levels of CXCL9 and CCL17 and clinical parameters

Judging from the comparison between the decline group and the stable group, and the logistic regression analysis, we concluded that a serum profile of lower CXCL9, in combination with higher CCL17, was an important predictor of worsening lung function. Next, we analyzed the correlation between the serum levels of CXCL9 and CCL17 and the clinical parameters of chronic bird-related HP (Table 4). Serum CXCL9 was significantly but weakly correlated with age (*r* = 0.255, *P* = 0.048), A-aDO_2_ (*r* = 0.381, *P* = 0.003), DL_CO_ (*r* = -0.268, *P* = 0.044), ΔVC (*r* = 0.307, *P* = 0.034), Δ%VC (*r* = 0.286, *P* = 0.049), and BALF lymphocytes (*r* = 0.352, *P* = 0.019) and moderately correlated with BALF macrophages (*r* = -0.428, *P* = 0.004). Serum CCL17 was significantly but weakly correlated with Δ%VC (*r* = -0.398, *P* = 0.005) and moderately correlated with ΔVC (*r* = -0.414, *P* = 0.003) and BALF macrophages (*r* = 0.438, *P* = 0.003). Serum CXCL9 also showed significant but weak correlations with the GGO score (*r* = 0.282, *P* = 0.035) and centrilobular nodules (*r* = -0.305, *P* = 0.023). Serum CCL17 also showed a significant but weak correlation with consolidation (*r* = 0.265, *P* = 0.048) and moderate correlations with the fibrosis score (*r* = 0.460, *P* < 0.001) and TBE (*r* = 0.473, *P* < 0.001) (Table 5).

**Table 4:** Relationship between the serum levels of CXCL9 and CCL17 and clinical parameters.

	CXCL9		CCL17	
	*r*	*P*	*r*	*P*
Age	0.255	0.048*	0.078	0.550
Pack years	0.171	0.186	0.043	0.744
Serum CRP, mg/dl	0.165	0.203	0.071	0.585
Serum KL-6, U/ml	0.140	0.282	-0.010	0.938
A-aDO_2_, mmHg	0.381	0.003**	-0.225	0.082
PFTs				
VC, L	-0.232	0.072	-0.076	0.560
%VC	-0.069	0.600	-0.249	0.053
FEV_1_/FVC	-0.050	0.699	0.202	0.119
DL_CO_, ml/min/mmHg	-0.268	0.044*	-0.215	0.109
%DL_CO_	-0.229	0.087	-0.231	0.084
ΔVC, L	0.307	0.034*	-0.414	0.003**
Δ%VC	0.286	0.049*	-0.398	0.005**
Δ DL_CO_, ml/min/mmHg	0.074	0.659	-0.301	0.066
Δ %DL_CO_	-0.001	0.994	-0.268	0.104
BALF				
Total cell counts, 10^5^/ml	0.090	0.586	-0.225	0.169
Macrophages, %	-0.428	0.004**	0.438	0.003**
Lymphocytes, %	0.352	0.019*	-0.153	0.323
Neutrophils, %	0.210	0.176	-0.235	0.129
Eosinophils, %	0.074	0.639	-0.008	0.957
CD4/CD8 ratio	0.314	0.075	0.190	0.290

* *P* < 0.05.

** *P* < 0.01.

HP: hypersensitivity pneumonitis, A-aDO_2_: alveolar-arterial oxygen difference, KL-6: Krebs von den Lungen 6, PFTs: pulmonary function tests, VC: vital capacity, DL_co_: diffusing capacity of the lung for carbon monoxide, BALF: bronchoalveolar lavage fluid.

**Table 5:** Relationship between the serum levels of CXCL9 and CCL17 and HRCT findings.

	CXCL9		CCL17	
	*r*	*P*	*r*	*P*
GGO score	0.282	0.035*	-0.021	0.876
Fibrosis score	-0.045	0.743	0.460	<0.001**
Reticulation, %	0.183	0.177	0.286	0.033*
Centrilobular nodules, %	-0.305	0.023*	-0.046	0.736
Consolidation, %	-0.138	0.312	0.265	0.048*
Emphysema, %	0.201	0.137	-0.021	0.878
TBE grade	-0.009	0.950	0.473	<0.001**

* *P* < 0.05.

** *P* < 0.01.

HP: hypersensitivity pneumonitis, HRCT: high-resolution computed tomography, GGO: ground grass opacity, TBE: traction bronchiectasis.

### Relationship between CXCL9 and CCL17 BALF levels and clinical parameters

We also analyzed the correlation between the BALF levels of CXCL9 and CCL17 and clinical parameters in chronic bird-related HP (S4 Table). BALF CXCL9 showed significant moderate correlation with serum CXCL9 (*r* = 0.452, *P* = 0.011), serum CCL17 (*r* = -0.440, *P* = 0.013), serum KL-6 (*r* = 0.469, *P* = 0.008), VC (*r* = -0.535, *P* = 0.002), %VC (*r* = -0.525, *P* = 0.002), DL_CO_ (*r* = -0.500, *P* = 0.005), %DL_CO_ (*r* = -0.433, *P* = 0.017), ΔVC (*r* = 0.434, *P* = 0.034), Δ%VC (*r* = 0.463, *P* = 0.023), BALF lymphocytes (*r* = 0.623, *P* < 0.001), and BALF neutrophils (*r* = 0.439, *P* = 0.013), and significant strong correlation with BALF macrophages (*r* = -0.751, *P* < 0.001). BALF CCL17 showed significant weak correlation with A-aDO_2_ (*r* = -0.362, *P* = 0.045), %VC (*r* = -0.342, *P* = 0.060), FEV_1_/FVC (*r* = 0.399, *P* = 0.026), centrilobular nodules (*r* = 0.398, *P* = 0.033), and TBE grade (*r* = 0.386, *P* = 0.039) and significant moderate correlation with BALF neutrophils (*r* = 0.412, *P* = 0.021), GGO score (*r* = -0.421, *P* = 0.023) and consolidation (*r* = 0.634, *P* < 0.001).

### Cluster analysis using serum levels of CXCL9 and CCL17

**Fig 1:**
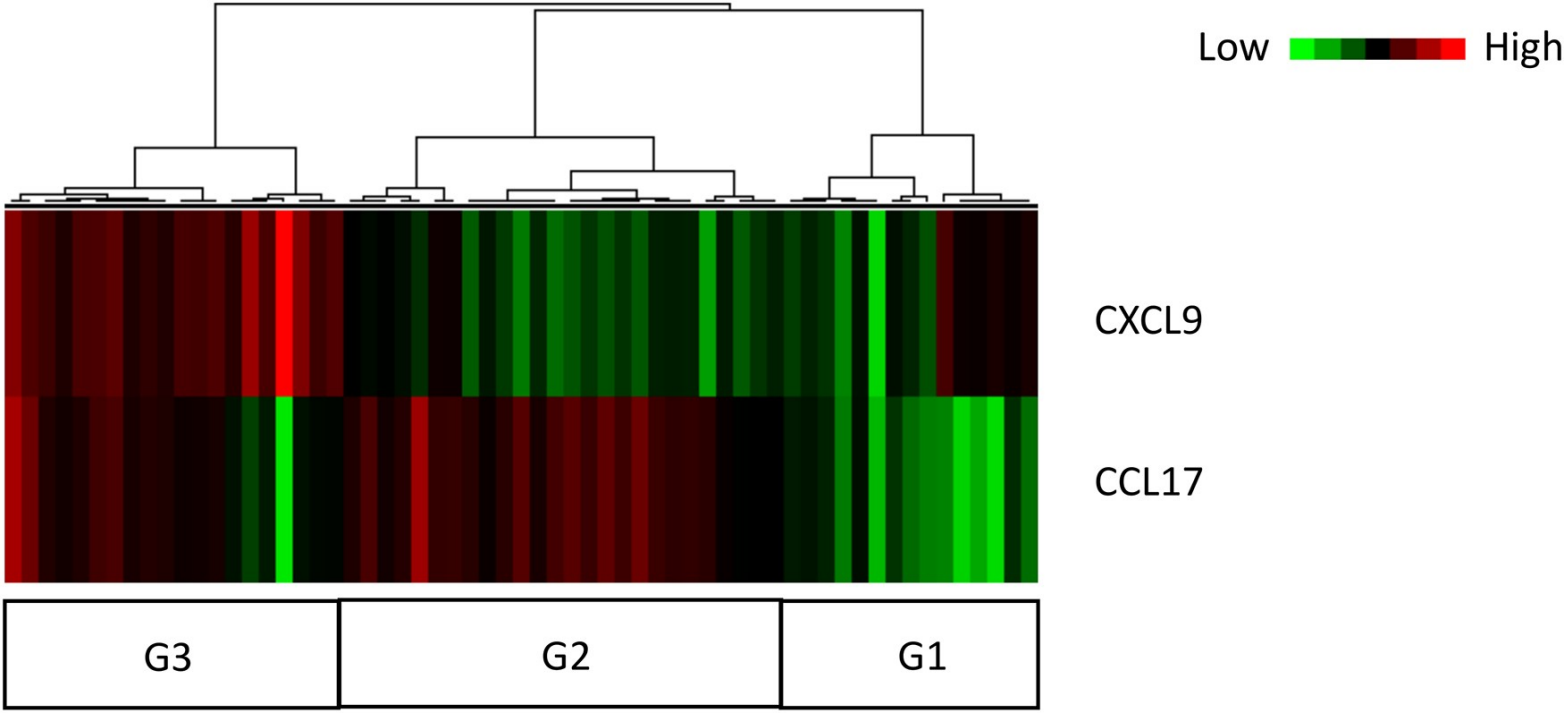
Unsupervised hierarchical clustering analysis of CXCL9 and CCL17 serum levels. Cluster analysis was performed by complete linkage based on Euclidean distance. Red colors represent high cytokines levels and green colors represent low cytokines levels, respectively. By clustering analysis, the patients with chronic bird-related HP were divided into three groups (G1, G2 and G3).

**Fig 2:**
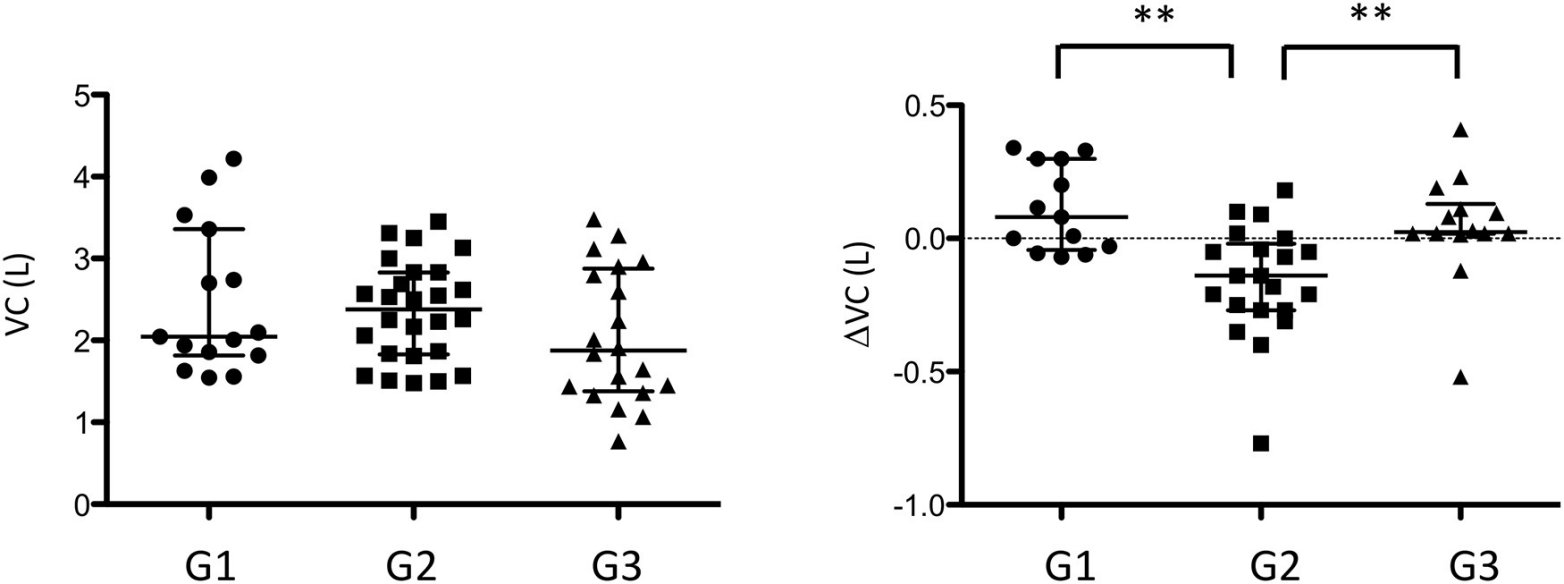
VC and ΔVC were compared among three groups (G1, G2 and G3). VC: vital capacity. The lines represent median and interquartile range. **: *P* < 0.01.

We also examined the relationship of CXCL9 and CCL17 serum levels by cluster analysis (Fig 1, Table 6). The patients with chronic bird-related HP were divided into three groups: G1 patients, with a serum profile of lower CXCL9 and lower CCL17; G2 patients, with lower CXCL9 and higher CCL17; and G3 patients, with higher CXCL9 and higher CCL17. The VC decline was significantly greater in G2 patients compared to G1 and G3 patients (Fig 2). BALF lymphocytes were significantly higher in G3 patients compared to G2 patients.

**Table 6:** Comparison of clinical findings among the three groups identified in cluster analysis.

	G1	G2	G3
Number	15	26	20
Age, yr.	60.0	62.0	69.0
Gender (Male / Female)	6 / 9	18 / 8	9 / 11
Pack years	0.0 (0.0–2.8)	11.0 (0.0–31.3)	0.0 (0.0–27.3)
Treatment during first 6 months	8	5	7
Serum biomarkers			
CXCL9, pg/ml	18.0 (12.3–23.7) ^§^	14.5 (9.8–17.4) ^§^	39.9 (32.8–46.0)
CCL17, pg/ml	135.0 (71.0–306.4) ^§^ ^¶^	726.0 (569.9–916.0) *	546.8 (378.7–642.6)
KL-6, U/ml	728 (345–2292)	1005 (536–1342) *	1518 (1032–2076)
HRCT findings			
GGO score	1.00 (0.73–1.44)	0.75 (0.42–1.06)	1.13 (0.56–1.71)
Fibrosis score	1.00 (0.29–1.25) ^#^	1.33 (1.00–1.58)	1.33 (0.79–1.52)
Reticulation, %	5.21 (0.73–7.19)	5.21 (3.85–6.67)	5.83 (3.13–9.79)
Centrilobular nodules, %	0.00 (0.00–1.15)	0.00 (0.00–1.56)	0.00 (0.00–0.10)
Consolidation, %	0.00 (0.00–2.71)	1.46 (0.42–2.81) *	0.42 (0.00–1.25)
Emphysema, %	0.00 (0.00–0.21)	0.00 (0.00–0.42)	0.00 (0.00–5.83)
TBE grade	0.33 (0.06–0.63) * ^#^	0.63 (0.42–0.90)	0.67 (0.33–0.85)
PFTs			
VC, L	2.05 (1.82–3.36)	2.38 (1.83–2.83)	1.88 (1.38–2.88)
%VC	82.4 (68.2–94.6)	76.1 (65.8–83.1)	73.4 (61.4–84.3)
DL_CO_, ml/min/mmHg	11.77 (10.28–13.22) ^§^ *	9.95 (6.40–12.47)	6.64 (4.67–9.49)
%DL_CO_	63.7 (50.7–74.7) ^§^	56.6 (39.4–66.1)	42.7 (33.3–55.4)
ΔVC, L	0.08 (-0.04–0.30) ^¶^	-0.14 (-0.27–-0.02) ^§^	0.03 (0.02–0.13)
Δ%VC	3.3 (-1.6–10.1) ^¶^	-5.5 (-8.6–-0.3) ^§^	1.4 (0.5–5.7)
Δ DL_CO_, ml/min/mmHg	0.41 (-0.85–1.09)	-0.36 (-1.74–0.58)	-0.30 (-0.47–0.54)
Δ %DL_CO_	3.5 (-4.9–5.1)	-1.3 (-8.2–2.2)	-2.9 (-4.9–3.2)
BALF			
Macrophages, %	65.1 (32.3–84.3) ^#^	84.4 (73.2–92.1) ^§^	60.6 (37.0–80.0)
Lymphocytes, %	14.0 (6.0–42.4)	11.6 (3.6–20.4) ^§^	23.4 (12.3–57.8)
Neutrophils, %	13.1 (0.7–29.1)	1.2 (0.6–3.6)	1.6 (0.8–6.0)
Eosinophils, %	0.8 (0.0–1.3)	1.3 (0.6–1.6)	1.0 (0.0–2.6)
CD4/8 ratio	1.7 (0.8–7.9)	3.0 (2.0–4.8)	5.3 (2.7–12.7)

Data are given as numbers or medians and interquartiles.

^§^: *P* < 0.01 versus G3.

^¶^: *P* < 0.01 versus G2.

*: *P* < 0.05 versus G3.

^#^: *P* < 0.05 versus G2.

KL-6: Krebs von den Lungen 6, HRCT: high-resolution computed tomography, GGO: ground grass opacity, TBE: traction bronchiectasis, BALF: bronchoalveolar lavage fluid, PFTs: pulmonary function tests, VC: vital capacity, DL_co_: diffusing capacity of the lung for carbon monoxide.

### ROC analysis to define the cutoff value of predictors of lung function decline

The results of the cluster analysis suggested that a chemokine profile of lower CXCL9 and higher CCL17 in serum was related to a decline of VC. We therefore conducted an ROC analysis and defined the cutoff value for the CCL17 to CXCL9 ratio (CCL17/CXCL9) in serum with chronic bird-related HP between the decline and stable groups. The AUC and optimal cutoff value were 0.849, and 24.8, respectively. The sensitivity and specificity at this cutoff value were 70.6%, and 92.9%, respectively. We verified this result using a leave-one-out cross-validation. The AUC and optimal cutoff values were 0.849 ± 0.008, and 25.0 ± 1.0, respectively (mean ± standard deviation). From this result, we concluded that the optimal cutoff value could be accepted.

Survival and free from AE in chronic bird-related HP patients divided into the higher-CCL17/CXCL9 group (CCL17/CXCL9 ≥ 24.8) and lower-CCL17/CXCL9 group (CCL17/CXCL9 < 24.8) were analyzed using Kaplan-Meier survival curves (S2 Fig). The log-rank test showed no significant differences between CCL17/CXCL9 and survival time (*P* = 0.100). The log-rank test also showed no significant differences between CCL17/CXCL9 and the duration free from AE (*P* = 0.290).

### Analysis of the difference in corticosteroid efficacy

We analyzed the difference in corticosteroid efficacy among the G1, G2, and G3 patients. No significant difference in ΔVC ^treatment^ was found among the three groups. On the other hand, ΔKL-6 ^treatment^ was significantly smaller in the G3 patients compared to the G2 patients (S5 Table). A significant positive correlation was also found between CCL17/CXCL9 and ΔKL-6 ^treatment^ (*r* = 0.361, *P* = 0.024).

## Discussion

In this study we investigated the predictors of the phenotypes with worsening lung function. Judging from the comparisons between the decline group and stable group and logistic regression analysis, we concluded that a chemokine profile of lower CXCL9 and higher CCL17 in serum was an important predictor of the phenotypes with worsening lung function. We confirmed these results using cluster analysis. Furthermore, a CCL17/CXCL9 ratio of above 24.8 in the serum of chronic bird-related HP patients was associated with VC decline at 6 months from the first admission. This is the first report to demonstrate the predictors of the phenotypes with worsening lung function in patients with chronic bird-related HP.

CCL17 is a Th2 cytokines mainly produced by epithelial cells [17]. Previous reports suggested that CCL17 and the ligand CCR4 contribute to development of pulmonary fibrosis by inducing infiltration of alveolar macrophages and Th2 cells in a bleomycin mouse model. [18]. It was also reported that the elevated CCL17 levels in BALF were associated with poor outcomes in patients with IPF [19]. Our group previously demonstrated that the expression of CCL17 and CCR4 were induced at the site of fNSIP and UIP lesions [9]. In the present study, the CCL17serum levels showed significant positive correlations with the fibrosis score and TBE grade, negative correlation with Δ%VC, and moderate correlation with ΔVC. Moreover, the BALF levels of CCL17 showed significant negative correlation with %VC, GGO score and TBE grade. These results might indicate that Th2 predominant immune response plays an important role in the development of lung fibrosis in patients with chronic HP.

On the other hand, our group also demonstrated that higher BALF neutrophil counts were observed in patients with a greater extent of fibroblastic foci compared to patients with a lesser extent of fibroblastic foci [5]. In the present study, the BALF levels of CCL17 also correlated with BALF neutrophils, supporting the relation between CCL17 and pathogenesis of pulmonary fibrosis.

CXCL9 is an IFN-γ-inducible chemokine, a ligand for CXCR3, and a chemokine frequently associated with Th1 diseases. Nance et al. reported that IFN-γ-deficient mouse showed lower CXCL9 production and fewer granuloma formation in the HP induction model [20]. On the bleomycin model, it was also reported that fibrogenesis is increased in CXCR3-deficient mice, suggesting Th1 inflammation plays a protective role [21]. The CXCL9 pathway suppresses collagen production in LX-2 cells, and CXCR3-deficient mice also demonstrated the anti-fibrotic effect of Th1 inflammation in liver fibrosis [22]. It was reported that CXCL9 reduced TGF-β1-induced phosphorylation of Smad2 and Smad3, and it abrogated the TGF-β1-induced epithelial-to-mesenchymal transition in human alveolar epithelial cells [23]. CXCL9 and CXCL10 were highly expressed in lung specimens and BALF in patients with HP [24, 25]. We also previously reported that chronic HP and acute HP patients with cNSIP/OP pattern showed higher CXCL10 levels and BALF lymphocytes compared to the UIP and the fNSIP pattern that is generally considered as an advanced fibrosis stage. By contrast, BALF macrophages were significantly lower in patients with the cNSIP/OP pattern compared to the UIP and fNSIP patterns [9]. In the present study, the CXCL9 serum levels had a significant positive correlation with BALF lymphocytes and the GGO score, and had a negative correlation with BALF macrophages. Serum CXCL9 also showed significant positive correlations with ΔVC and Δ%VC. These results and previous evidence suggest that CXCL9 plays a suppressive role to the progress of fibrosis in patients with chronic bird-related HP. In the comparison between decline and stable groups, not only BALF lymphocytes but also BALF macrophages had significant differences. In the previous reports of chronic HP, BALF lymphocytes had lower in the UIP-like patient they exhibited the worst survival rate [3, 26]. Those evidences suggest BALF lymphocytes were important factors in the pathology of chronic HP. The percentages of BALF macrophages might be affected that of BALF lymphocytes. In the present study, CXCL9 levels also had a significant positive correlation with neutrophils in BALF. It is known that neutrophils and T cells produce IFN-γ [27]. IFN-gamma resulted in an increase in CXCL9 mRNA expression by human alveolar epithelial type II or A549 cells [28]. In our previous study, we demonstrated that IL-17A and neutrophils were crucial for the development of pulmonary inflammation in murine models of acute HP [29]. Those inflammatory responses may contribute to CXCL9 production in HP patients.

Serum CXCL9, meanwhile, showed a weak negative correlation with the centrilobular nodules in the present study. A previous report has identified centrilobular nodules as the predominant finding in acute HP rather than chronic HP [6]. Our result on the correlation between serum CXCL9 and CT findings on centrilobular nodules in the present study directly oppose those of the previous study. However the proportion of lung parenchyma in centrilobular nodules in the present study was too small to compare the previous study, the analysis related to centrilobular nodules might be insufficient. Further study will be needed to clarify the relation between serum CXCL9 and centrilobular nodules on CT.

As discussed earlier, CCL17 plays an important role in the pathogenesis of pulmonary fibrosis and CXCL9 may have an antifibrotic effect in chronic bird-related HP. We demonstrated that a chemokine profile of lower CXCL9 and higher CCL17 in serum was an important predictor of the phenotypes with worsening lung function.

In this study we divided patients into three groups according to their CCL17 and CXCL9 profiles and analyzed the effect of corticosteroid on each group. The decline of serum KL-6 level was reported as the useful predictive marker of high-dose corticosteroids on patients with rapidly progressing IPF [30]. In patients with drug induced pneumonitis, serum KL-6 levels were also reported as the marker of response to withdrawal of the implicated drug and/or corticosteroid therapy [31]. In previous study of patients with chronic HP, serum KL-6 level at 1 month after the treatment of prednisolone was significantly lower than those measured at the start of the treatment. In that study, the serum KL-6 level was significantly higher during episode of AE [32]. Though there was no established marker of treatment in chronic HP, we evaluated the efficacy of corticosteroid using serum KL-6 level based on these results. The corticosteroid-dependent decrease of serum KL-6 was significantly greater in patients of the G3 type, characterized by higher CXCL9 and higher CCL17, compared to the G2 type, characterized by lower CXCL9 and higher CCL17. There was also a significant positive correlation between the CCL17/CXCL9 ratio and ΔKL-6 ^treatment^. Thus, the G3 patients might tend to improve with corticosteroid treatment. In the previous report of IPF, patients treated with corticosteroid exhibited higher CXCR3 in BALF CD4 lymphocytes [33]. Patients with higher CXCL9 might be more influenced by CXCL9 and CXCR3 than patients with lower CXCL9. BALF lymphocytes were considered to be one of the inflammatory features in HP and use of corticosteroid or immunosuppressants might be reasonable in patients with inflammatory features [34]. As the G3 patients had higher BALF lymphocytes, corticosteroid might be effective to the G3 patients.

We analyzed cumulative survival and AE-free interval between higher and lower CCL17/CXCL9 group. There was no statistical significance of cumulative survival and AE-free intervals. Treatment during the follow-up period may affect this result.

This study had several limitations. First, it was performed in only a single center, and one with a referral bias, as our hospital is a clinical center for chronic HP. Second, the subjects of this study were limited to bird-related HP, the most prevalent form of HP in Japan. Third, the sample size of our analysis of the efficacy of corticosteroid was relatively small and the efficacy of antifibrotic agent was not analyzed. All of those limitations are based on the low prevalence rate of HP; however, we will provide answers for those issues in future studies.

In conclusion, we demonstrated that HP consisted of different inflammatory-endotypes, and proposed that the serum CCL17/CXCL9 ratio was an important biomarker for worsening lung function in patients with chronic bird-related HP.

## Supporting information

S1 Fig Serum levels of CXCL9, CCL17, and KL-6 in patients with chronic bird-related hypersensitivity pneumonitis and healthy volunteers. CBRHP: chronic bird-related hypersensitivity pneumonitis, n = 61, HV: healthy volunteers, n = 50. The lines represent median and interquartile range. **: P < 0.01.
(TIF)

S2 Fig Kaplan-Meier curves for survival (A) and acute exacerbation-free interval (B) in chronic bird-related hypersensitivity pneumonitis. Solid line represents higher-CCL17/CXCL9 group (serum CCL17/CXCL9 levels ≥ 24.8) dotted line represents lower-CCL17/CXCL9 group (< 24.8). (A) P = 0.100, (B) P = 0.290, log rank test.
(TIF)

S1 Method The diagnostic criteria for chronic HP. (DOCX)

S1 Table BALF profiles. BALF profiles in chronic bird-related HP.
(DOCX)

S2 Table HRCT findings. (DOCX)

S3 Table Inter-observer correlation in HRCT findings. (DOCX)

S4 Table Relationship between the BALF levels of CXCL9 and CCL17 and clinical parameters. (DOCX)

S5 Table Analysis of the difference in corticosteroid efficacy. (DOCX)

## References

[1] Fink JN. Hypersensitivity pneumonitis. J Allergy Clin Immunol. 1984;74(1):1–10. 6429227 10.1016/0091-6749(84)90077-0

[2] Miyazaki Y, Tsutsui T, Inase N. Treatment and monitoring of hypersensitivity pneumonitis. Expert Rev Clin Immunol. 2016;12(9):953–62. 10.1080/1744666X.2016.1182426 27117830

[3] Ohtani Y, Saiki S, Kitaichi M, Usui Y, Inase N, Costabel U, et al Chronic bird fancier’s lung: histopathological and clinical correlation. An application of the 2002 ATS/ERS consensus classification of the idiopathic interstitial pneumonias. Thorax. 2005;60(8):665–71. 10.1136/thx.2004.027326 16061708 PMC1747497

[4] Miyazaki Y, Tateishi T, Akashi T, Ohtani Y, Inase N, Yoshizawa Y. Clinical predictors and histologic appearance of acute exacerbations in chronic hypersensitivity pneumonitis. Chest. 2008;134(6):1265–70. 10.1378/chest.08-0866 18689595

[5] Chiba S, Tsuchiya K, Akashi T, Ishizuka M, Okamoto T, Furusawa H, et al Chronic Hypersensitivity Pneumonitis With a Usual Interstitial Pneumonia-Like Pattern: Correlation Between Histopathologic and Clinical Findings. Chest. 2016;149(6):1473–81. 10.1016/j.chest.2015.12.030 26836921

[6] Tateishi T, Ohtani Y, Takemura T, Akashi T, Miyazaki Y, Inase N, et al Serial high-resolution computed tomography findings of acute and chronic hypersensitivity pneumonitis induced by avian antigen. J Comput Assist Tomogr. 2011;35(2):272–9. 10.1097/RCT.0b013e318209c5a6 21412103

[7] Tsutsui T, Miyazaki Y. The amount of avian antigen in household dust predicts the prognosis of chronic bird-related hypersensitivity pneumonitis. Ann Am Thorac Soc. 2015;12(7):1013–21. 10.1513/AnnalsATS.201412-569OC 26010749

[8] Sema M, Miyazaki Y. Environmental levels of avian antigen are relevant to the progression of chronic hypersensitivity pneumonitis during antigen avoidance. Immun Inflamm Dis. 2018;6(1):154–62. 10.1002/iid3.202 29168324 PMC5818447

[9] Kishi M, Miyazaki Y, Jinta T, Furusawa H, Ohtani Y, Inase N, et al Pathogenesis of cBFL in common with IPF? Correlation of IP-10/TARC ratio with histological patterns. Thorax. 2008;63(9):810–6. 10.1136/thx.2007.086074 18276722

[10] Yoshizawa Y, Ohtani Y, Hayakawa H, Sato A, Suga M, Ando M. Chronic hypersensitivity pneumonitis in Japan: a nationwide epidemiologic survey. J Allergy Clin Immunol. 1999;103:315–20. 10.1016/s0091-6749(99)70507-5 9949324

[11] Ohtani Y, Kojima K, Sumi Y, Sawada M, Inase N, Miyake S, et al Inhalation provocation tests in chronic bird fancier’s lung. Chest. 2000;118(5):1382–9. 10.1378/chest.118.5.1382 11083690

[12] Kondoh Y, Taniguchi H, Kawabata Y, Yokoi T, Suzuki K, Takagi K. Acute exacerbation in idiopathic pulmonary fibrosis. Analysis of clinical and pathologic findings in three cases. Chest. 1993;103(6):1808–12. 10.1378/chest.103.6.1808 8404104

[13] Kazerooni EA, Martinez FJ, Flint A, Jamadar DA, Gross BH, Spizarny DL, et al Thin-section CT obtained at 10-mm increments versus limited three-level thin-section CT for idiopathic pulmonary fibrosis: correlation with pathologic scoring. AJR Am J Roentgenol. 1997;169(4):977–83. 10.2214/ajr.169.4.9308447 9308447

[14] Walsh SL, Sverzellati N, Devaraj A, Keir GJ, Wells AU, Hansell DM. Connective tissue disease related fibrotic lung disease: high resolution computed tomographic and pulmonary function indices as prognostic determinants. Thorax. 2014;69(3):216–22. 10.1136/thoraxjnl-2013-203843 24127020

[15] Roland M, Weycker D, Albera C, Bradford WZ, Costabel U, Kartashov A, et al Forced vital capacity in patients with idiopathic pulmonary fibrosis: test properties and minimal clinically important difference. Am J Respir Crit Care Med. 2011;184(12):1382–9. 10.1164/rccm.201105-0840OC 21940789

[16] Zappala CJ, Latsi PI, Nicholson AG, Colby TV, Cramer D, Renzoni EA, et al Marginal decline in forced vital capacity is associated with a poor outcome in idiopathic pulmonary fibrosis. Eur Respir J. 2010;35(4):830–6. 10.1183/09031936.00155108 19840957

[17] Sekiya T, Miyamasu M, Imanishi M, Yamada H, Nakajima T, Yamaguchi M, et al Inducible expression of a Th2-type CC chemokine thymus- and activation-regulated chemokine by human bronchial epithelial cells. J Immunol. 2000;165(4):2205–13. 10.4049/jimmunol.165.4.2205 10925308

[18] Belperio JA, Dy M, Murray L, Burdick MD, Xue YY, Strieter RM, et al The role of the Th2 CC chemokine ligand CCL17 in pulmonary fibrosis. J Immunol. 2004;173(7):4692–8. 10.4049/jimmunol.173.7.4692 15383605

[19] Shinoda H, Tasaka S, Fujishima S, Yamasawa W, Miyamoto K, Nakano Y, et al Elevated CC chemokine level in bronchoalveolar lavage fluid is predictive of a poor outcome of idiopathic pulmonary fibrosis. Respiration. 2009;78(3):285–92. 10.1159/000207617 19270434

[20] Nance S, Cross R, Fitzpatrick E. Chemokine production during hypersensitivity pneumonitis. Eur J Immunol. 2004;34(3):677–85. 10.1002/eji.200324634 14991597

[21] Jiang D, Liang J, Hodge J, Lu B, Zhu Z, Yu S, et al Regulation of pulmonary fibrosis by chemokine receptor CXCR3. J Clin Invest. 2004;114(2):291–9. 10.1172/JCI16861 15254596 PMC449741

[22] Wasmuth HE, Lammert F, Zaldivar MM, Weiskirchen R, Hellerbrand C, Scholten D, et al Antifibrotic effects of CXCL9 and its receptor CXCR3 in livers of mice and humans. Gastroenterology. 2009;137(1):309–19. 10.1053/j.gastro.2009.03.053 19344719 PMC2892869

[23] O’Beirne SL, Walsh SM, Fabre A, Reviriego C, Worrell JC, Counihan IP, et al CXCL9 Regulates TGF-beta1-Induced Epithelial to Mesenchymal Transition in Human Alveolar Epithelial Cells. J Immunol. 2015;195(6):2788–96. 10.4049/jimmunol.1402008 26268659 PMC4777321

[24] Selman M, Pardo A, Barrera L, Estrada A, Watson SR, Wilson K, et al Gene expression profiles distinguish idiopathic pulmonary fibrosis from hypersensitivity pneumonitis. Am J Respir Crit Care. 2006;173(2):188–98. 10.1164/rccm.200504-644OC PMC2662988 16166619

[25] Agostini C, Calabrese F, Poletti V, Marcer G, Facco M, Miorin M, et al CXCR3/CXCL10 interactions in the development of hypersensitivity pneumonitis. Respir Res. 2005;6: 20 10.1186/1465-9921-6-20 15725351 PMC554979

[26] Gaxiola M, Buendia I, Mejia M, Carrillo G, Estrada A, Navarro MC, et al Morphologic diversity of chronic pigeon breeder’s disease: clinical features and survival. Respir med. 2011;105: 608–614. 10.1016/j.rmed.2010.11.026 21167698

[27] Nance S, Cross R, Yi AK, Fitzpatrick EA. IFN-gamma production by innate immune cells is sufficient for development of hypersensitivity pneumonitis. Eur J Immunol. 2005;35(6):1928–38. 10.1002/eji.200425762 15884056

[28] Pechkovsky DV, Goldmann T, Ludwig C, Prasse A, Vollmer E, Müller-Quernheim J, et al CCR2 and CXCR3 agonistic chemokines are differently expressed and regulated in human alveolar epithelial cells type II. Respir Res. 2005 7 20;6:75 10.1186/1465-9921-6-75 16033640 PMC1185567

[29] Ishizuka M, Miyazaki Y, Masuo M, Suhara K, Tateishi T, Yasui M, et al Interleukin-17A and Neutrophils in a Murine Model of Bird-Related Hypersensitivity Pneumonitis. PloS one. 2015;10(9): e0137978 10.1371/journal.pone.0137978 26367130 PMC4569356

[30] Yokoyama A, Kohno N, Hamada H, Sakatani M, Ueda E, Kondo K, et al Circulating KL-6 predicts the outcome of rapidly progressive idiopathic pulmonary fibrosis. Am J Respir Crit Care Med. 1998;158(5 Pt 1):1680–4. 10.1164/ajrccm.158.5.9803115 9817725

[31] Ohnishi H, Yokoyama A, Yasuhara Y, Watanabe A, Naka T, Hamada H, et al Circulating KL-6 levels in patients with drug induced pneumonitis. Thorax. 2003;58(10):872 10.1136/thorax.58.10.872 14514942 PMC1746480

[32] Okamoto T, Fujii M, Furusawa H, Tsuchiya K, Miyazaki Y, Inase N. The usefulness of KL-6 and SP-D for the diagnosis and management of chronic hypersensitivity pneumonitis. Respir Med. 2015;109(12):1576–81. 10.1016/j.rmed.2015.10.005 26481343

[33] Pignatti P, Brunetti G, Moretto D, Yacoub MR, Fiori M, Balbi B, et al Role of the chemokine receptors CXCR3 and CCR4 in human pulmonary fibrosis. Am J Respir Crit Care Med. 2006;173(3):310–7. 10.1164/rccm.200502-244OC 16239626

[34] Salisbury ML, Myers JL, Belloli EA, Kazerooni EA, Martinez FJ, Flaherty KR. Diagnosis and Treatment of Fibrotic Hypersensitivity Pneumonia. Where We Stand and Where We Need to Go. Am J Respir Crit Care Med. 2017;196(6):690–699. 10.1164/rccm.201608-1675PP 28002680 PMC5620675

