# Supplementary figures and images for: Serum CXCL9 and CCL17 as biomarkers of declining pulmonary function in chronic bird-related hypersensitivity pneumonitis
Source: PLoS One. 2019 Aug 1;14(8):e0220462. doi: 10.1371/journal.pone.0220462 (PMC6675044; doi:10.1371/journal.pone.0220462)

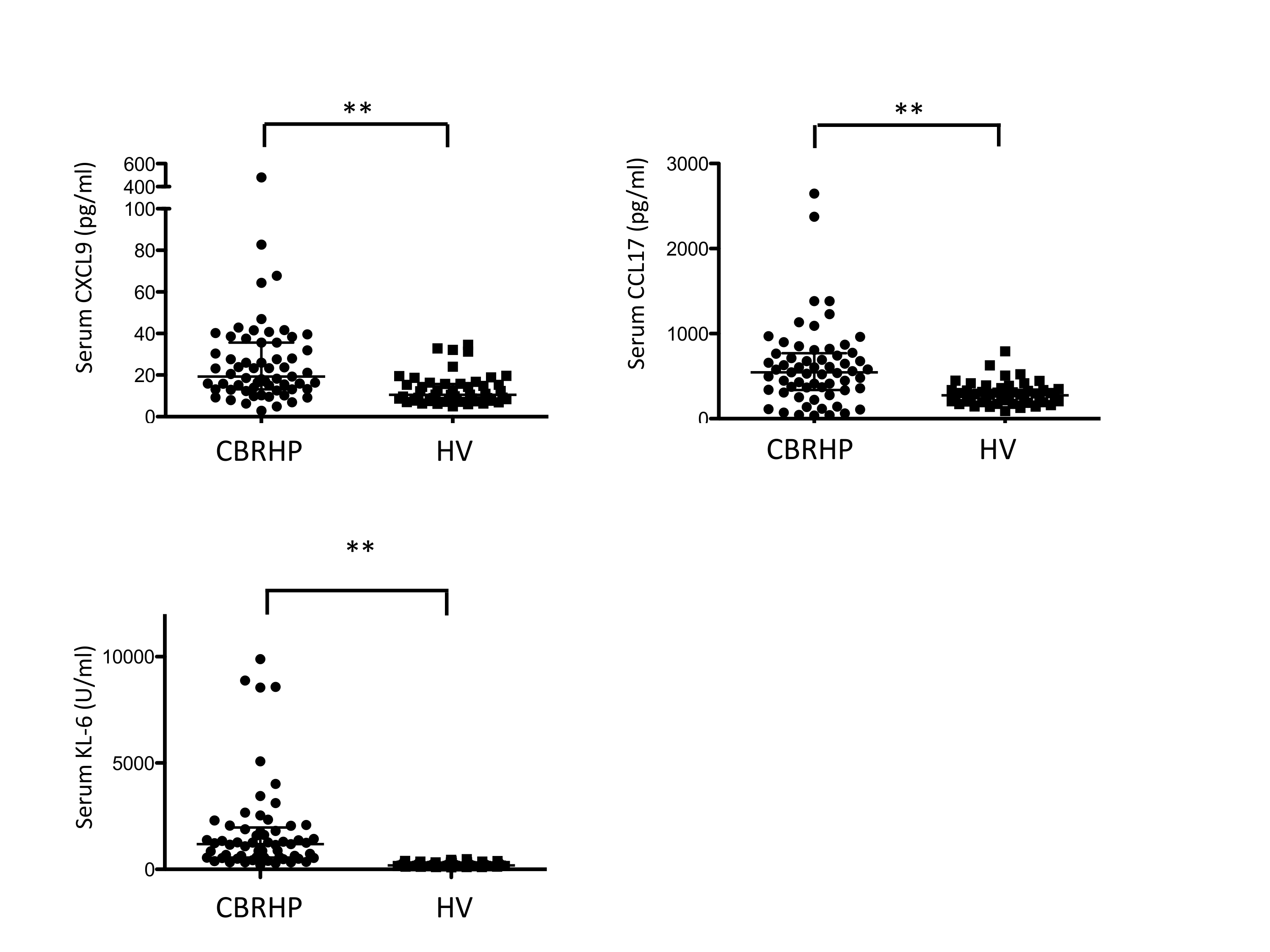

Supplement: S1 Fig — CBRHP: chronic bird-related hypersensitivity pneumonitis, n = 61, HV: healthy volunteers, n = 50. The lines represent median and interquartile range. **: P < 0.01. (TIF) [file pone.0220462.s001.tif]

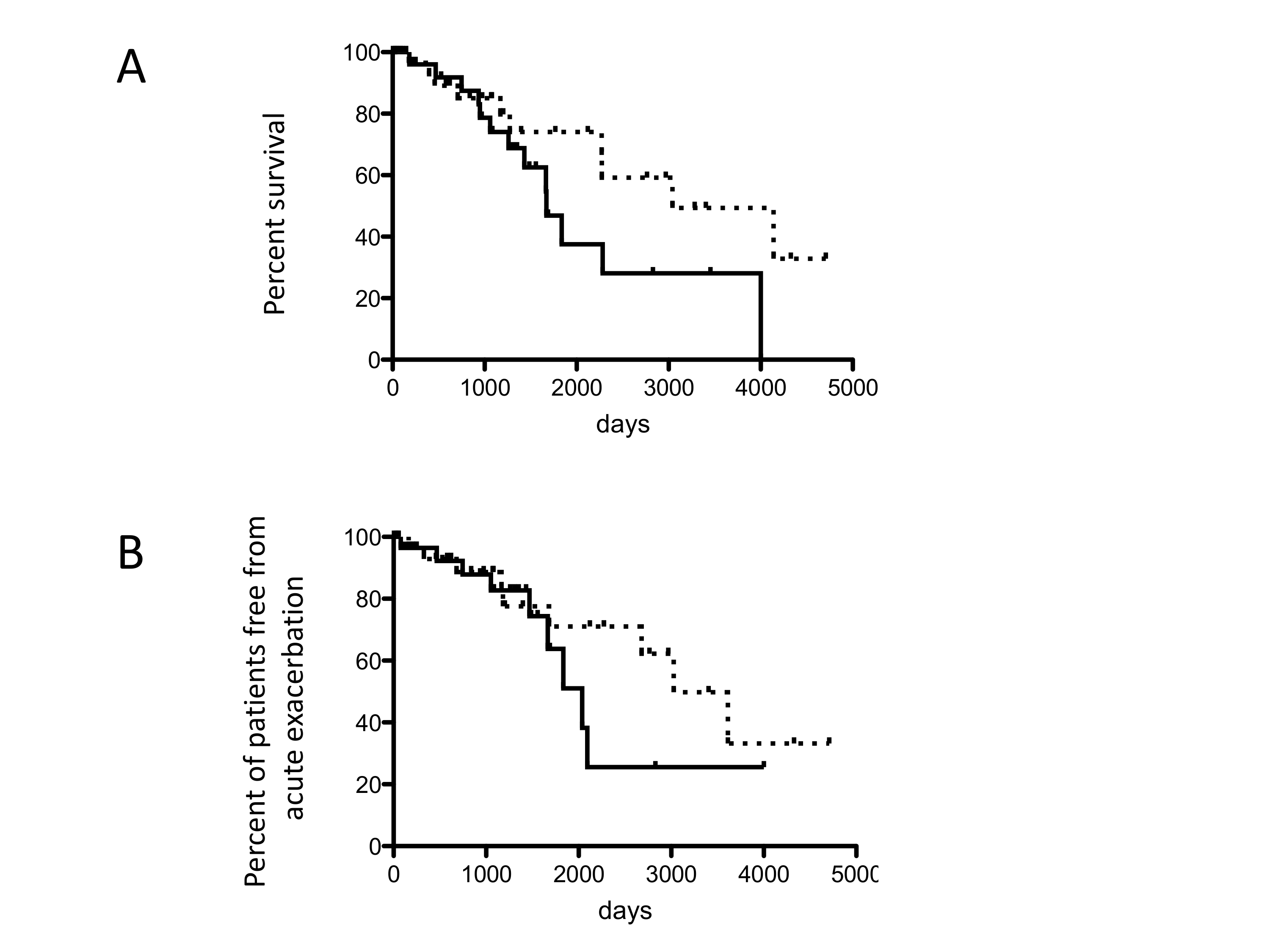

Supplement: S2 Fig — Solid line represents higher-CCL17/CXCL9 group (serum CCL17/CXCL9 levels ≥ 24.8) dotted line represents lower-CCL17/CXCL9 group (< 24.8). (A) P = 0.100, (B) P = 0.290, log rank test. (TIF) [file pone.0220462.s002.tif]
