# Supplementary material for: Serum CXCL9 and CCL17 as biomarkers of declining pulmonary function in chronic bird-related hypersensitivity pneumonitis
Source: PLoS One. 2019 Aug 1;14(8):e0220462. doi: 10.1371/journal.pone.0220462 (PMC6675044; doi:10.1371/journal.pone.0220462)
Supplement: S1 Method — (DOCX) [file pone.0220462.s003.docx]

**Online supplement**

**Methods**

**Criteria**

The diagnostic criteria for chronic HP were made based on the following Yoshizawa’s criteria: (1)

reproduction of symptoms of HP by an environmental provocation or laboratory-controlled

inhalation of the antigen; (2) positive results of antibodies and/or lymphocyte proliferation tests

to the antigen; (3) evidence of pulmonary fibrosis with or without granulomas; (4) finding of

honeycombing on resolution computed tomography; (5) progressive deterioration of restrictive

impairment of pulmonary function over one year; (6) more than six-month duration of respiratory

symptoms related to HP [including either (1) or (2), either (3) or (4), and either (5) or (6)] [1].

**Reference in supplementary materials**

1. Yoshizawa Y, Ohtani Y, Hayakawa H, Sato A, Suga M, Ando M. Chronic hypersensitivity pneumonitis in Japan: a nationwide epidemiologic survey. J Allergy Clin Immunol. 1999;　103:315-20.
