# Supplementary material for: Serum CXCL9 and CCL17 as biomarkers of declining pulmonary function in chronic bird-related hypersensitivity pneumonitis
Source: PLoS One. 2019 Aug 1;14(8):e0220462. doi: 10.1371/journal.pone.0220462 (PMC6675044; doi:10.1371/journal.pone.0220462)
Supplement: S1 Table — BALF profiles in chronic bird-related HP. (DOCX) [file pone.0220462.s004.docx]

**S1 Table** BALF profiles

|  | Chronic bird-related HP |
| --- | --- |
|  | n = 44 |
| Total cell counts, 10^5^/ml | 3.4 (2.6 - 5.6) |
| Macrophages, % | 74.6 (41.2 - 86.2) |
| Lymphocytes, % | 16.6 (7.6 - 38.7) |
| Neutrophils, % | 1.8 (0.7 - 10.4) |
| Eosinophils, % | 1.0 (0.0 - 1.6) |
| CD4/CD8 ratio | 3.3 (2.0 - 7.6) |
| BALF CXCL9, pg/ml^§^ | 8.1 (2.1 - 17.0) |
| BALF CCL17, pg/ml^§^ | 4.0 (2.0 - 6.8) |

Data are given as medians and interquartiles.

§: n=31

HP: hypersensitivity pneumonitis, BALF: bronchoalveolar lavage fluid.
