# Supplementary material for: Serum CXCL9 and CCL17 as biomarkers of declining pulmonary function in chronic bird-related hypersensitivity pneumonitis
Source: PLoS One. 2019 Aug 1;14(8):e0220462. doi: 10.1371/journal.pone.0220462 (PMC6675044; doi:10.1371/journal.pone.0220462)
Supplement: S2 Table — (DOCX) [file pone.0220462.s005.docx]

**S2 Table** HRCT findings

|  | Chronic bird-related HP |
| --- | --- |
| GGO score | 1.00 (0.50 - 1.42) |
| Fibrosis score | 1.21 (0.92 - 1.50) |
| Reticulation, % | 5.63 (3.33 - 7.40) |
| Centrilobular nodules, % | 0.00 (0.00 - 1.04) |
| Consolidation, % | 0.83 (0.00 - 2.50) |
| Emphysema, % | 0.00 (0.00 - 0.42) |
| TBE grade | 0.58 (0.33 - 0.83) |

Data are given as medians and interquartiles.

HP: hypersensitivity pneumonitis, HRCT: high-resolution computed tomography, GGO: ground grass opacity, TBE: traction bronchiectasis.
