# Supplementary material for: Serum CXCL9 and CCL17 as biomarkers of declining pulmonary function in chronic bird-related hypersensitivity pneumonitis
Source: PLoS One. 2019 Aug 1;14(8):e0220462. doi: 10.1371/journal.pone.0220462 (PMC6675044; doi:10.1371/journal.pone.0220462)
Supplement: S3 Table — (DOCX) [file pone.0220462.s006.docx]

**S3 Table** Inter-observer correlation in HRCT findings

|  | *r* | *P* |
| --- | --- | --- |
| GGO score | 0.720 | < 0.001 ^***^ |
| Fibrosis score | 0.807 | < 0.001 ^***^ |
| Reticulation, % | 0.716 | < 0.001 ^***^ |
| Centrilobular nodules, % | 0.521 | < 0.001 ^***^ |
| Consolidation, % | 0.710 | < 0.001 ^***^ |
| Emphysema, % | 0.893 | < 0.001 ^***^ |
| TBE grade | 0.717 | < 0.001 ^***^ |

^***^ *P* < 0.001

HRCT: high-resolution computed tomography, GGO: ground grass opacity, TBE: traction bronchiectasis.
