# Supplementary material for: Serum CXCL9 and CCL17 as biomarkers of declining pulmonary function in chronic bird-related hypersensitivity pneumonitis
Source: PLoS One. 2019 Aug 1;14(8):e0220462. doi: 10.1371/journal.pone.0220462 (PMC6675044; doi:10.1371/journal.pone.0220462)
Supplement: S4 Table — (DOCX) [file pone.0220462.s007.docx]

**S4 Table** Relationship between the BALF levels of CXCL9 and CCL17 and clinical parameters

|  | BALF CXCL9 | | BALF CCL17 | |
| --- | --- | --- | --- | --- |
|  | *r* | *P* | *r* | *P* |
| Serum CXCL9, pg/ml | 0.452 | 0.011^*^ | -0.265 | 0.149 |
| Serum CCL17, pg/ml | -0.440 | 0.013^*^ | 0.300 | 0.101 |
| Serum KL-6, U/ml | 0.469 | 0.008^**^ | -0.179 | 0.335 |
| A-aDO_2_, mmHg | 0.316 | 0.083 | -0.362 | 0.045^*^ |
| PFTs |  |  |  |  |
| VC, L | -0.535 | 0.002^**^ | -0.165 | 0.375 |
| %VC | -0.525 | 0.002^**^ | -0.342 | 0.060 |
| FEV_1_/FVC | 0.076 | 0.683 | 0.399 | 0.026^*^ |
| DL_CO_, ml/min/mmHg | -0.500 | 0.005^**^ | -0.136 | 0.475 |
| %DL_CO_ | -0.433 | 0.017^*^ | -0.280 | 0.135 |
| ΔVC, L | 0.434 | 0.034^*^ | -0.178 | 0.406 |
| Δ%VC | 0.463 | 0.023^*^ | -0.186 | 0.384 |
| Δ DL_CO_, ml/min/mmHg | 0.034 | 0.879 | -0.158 | 0.471 |
| Δ %DL_CO_ | 0.162 | 0.461 | -0.199 | 0.364 |
| BALF |  |  |  |  |
| Total cell counts, 10^5^/ml | 0.148 | 0.427 | 0.139 | 0.455 |
| Macrophages, % | -0.751 | < 0.001 ^***^ | 0.029 | 0.877 |
| Lymphocytes, % | 0.623 | < 0.001 ^***^ | -0.184 | 0.321 |
| Neutrophils, % | 0.439 | 0.013^*^ | 0.412 | 0.021^*^ |
| Eosinophils, % | 0.029 | 0.875 | 0.135 | 0.094 |
| CD4/CD8 ratio | 0.350 | 0.102 | 0.090 | 0.682 |
| HRCT findings |  |  |  |  |
| GGO score | 0.156 | 0.419 | -0.421 | 0.023^*^ |
| Fibrosis score | 0.235 | 0.220 | 0.203 | 0.292 |
| Reticulation, % | 0.352 | 0.061 | 0.222 | 0.247 |
| Centrilobular nodules, % | -0.031 | 0.872 | 0.398 | 0.033^*^ |
| Consolidation, % | 0.089 | 0.647 | 0.634 | < 0.001 ^***^ |
| Emphysema, % | -0.276 | 0.147 | -0.228 | 0.234 |
| TBE grade | 0.162 | 0.402 | 0.386 | 0.039^*^ |

^*^ *P* < 0.05, ^**^ *P* < 0.01, ^***^ *P* < 0.001.
