# Supplementary material for: Serum CXCL9 and CCL17 as biomarkers of declining pulmonary function in chronic bird-related hypersensitivity pneumonitis
Source: PLoS One. 2019 Aug 1;14(8):e0220462. doi: 10.1371/journal.pone.0220462 (PMC6675044; doi:10.1371/journal.pone.0220462)
Supplement: S5 Table — (DOCX) [file pone.0220462.s008.docx]

**S5 Table** Analysis of the difference in corticosteroid efficacy

|  | G1 | G2 | G3 |
| --- | --- | --- | --- |
| VC |  |  |  |
| Number | 13 | 11 | 6 |
| ΔVC ^treatment^, L | 0.11 (0.01 - 0.32) | 0.09 (0.03 - 0.31) | 0.06 (-0.22 - 0.28) |
| KL-6 |  |  |  |
| Number | 13 | 17 | 9 |
| ΔKL-6 ^treatment^, U/ml | -136 (-527 - 38) | 6 (-192 - 514) | -268 (-629 - -107) ^#^ |

Data are given as medians and interquartiles.

^#^: *P* < 0.05 versus G2.

KL-6: Krebs von den Lungen 6, VC: vital capacity.
